# EIOFX-DT: Leveraging graph centrality metrics for feature extraction and classification of viral genetic sequences

**DOI:** 10.1016/j.btre.2025.e00939

**Published:** 2025-11-27

**Authors:** Amin Khodaei, Zahra Pourabbas, Fatemeh Hashem-zadehdizajyekan, Erfan Esmaeili

**Affiliations:** a Faculty of Electrical & Computer Engineering, University of Tabriz, Tabriz, Iran; b Department of Computer Engineering, Tabriz Branch, Islamic Azad University, Tabriz, Iran

**Keywords:** Genetic data, Graph, Complex network, Eigen-vector centrality, Decision tree, Feature extraction

## Abstract

Many diseases have a genetic origin, and analyzing intracellular structures through genetic data yields specific features for the diagnosis and classification of viral disease samples. In this study, 30 types of viruses were analyzed using a graph-based approach on genetic data. Genetic data has been modeled in the form of genomic sequences at the nucleotide scale using the graph theory of complex networks concepts. Degree and eigenvector centrality metrics were employed to extract features. The decision tree was utilized as a machine learning classifier algorithm on the resulting feature space. The results, presented in the form of interpretable rules, enable the classification and identification of virus types from both a binary and multi-class perspective. The model achieved high accuracy and f1 score, which exceeded 99 % on >173,000 samples. Additionally, the feature extraction algorithm demonstrated robust performance across all datasets and classifiers.

## Highlights

• Modeling the structure of various viruses in the form of directed weighted graphs.
• Presenting a feature extraction algorithm based on complex networks metrics.
• The impact of the eigen-vector, input centrality and output centrality measures of specific nucleotide triplets within genes associated with particular virus types.
• The independence of the proposed approach's performance from the length or type of virus genetic data.
• Utilizing an interpretable classifier with the ability to relate findings to genetic concepts by extracting rules.

## Graphical abstract

**Figure:**
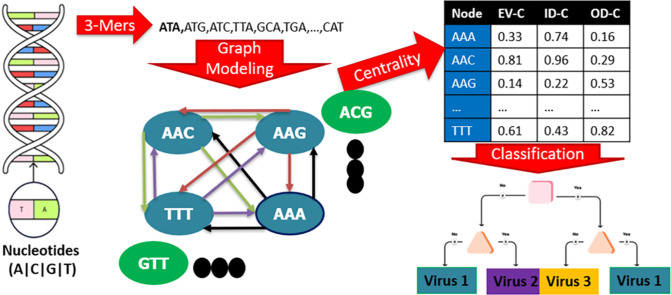

## 1 Introduction

Viruses are microscopic organisms that can infect hosts such as humans, plants and animals. They consist of a genomic segment enclosed in a protective shell. They are not made up of cells, they cannot reproduce without a host. In other words, they lack the full functionality that cells possess to replicate. Instead, they carry genetic instructions and take advantage of a host cell’s machinery to replicate themselves. The genome of DNA viruses includes early genes, which are involved in virus integration and replication, and late genes, which participate in the synthesis of viral capsid proteins and the assembly of virions [1].

Genetic changes in viruses occur through mutation and recombination, enabling rapid adaptation and the emergence of novel strains [1]. It is well established that accurate disease diagnosis can improve treatment efficacy in infected patients. Early identification enables targeted therapies, limits further spread, and improves patient outcomes, while also supporting broader public health efforts. In other words, the timely detection of newly emerging viruses is highly beneficial, as it facilitates early containment, the development of targeted vaccines, the advancement of effective treatment strategies, the expansion of virological knowledge, the enhancement of global preparedness, and the promotion of public safety and resilience [2,3].

**Fig. 1:**
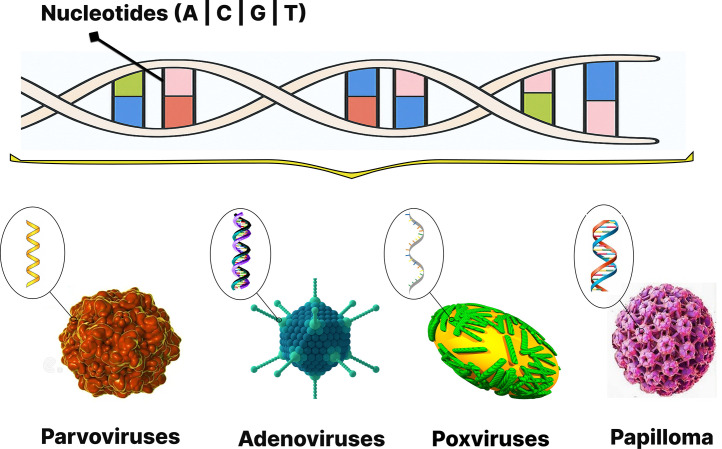
DNA Virus Structures.

The genetic structure inside cells consists of nucleotide sequences. The building blocks of nucleotide sequences, which include adenine (A), cytosine (C), guanine (G), and thymine (T) nucleotides. These nucleotides pair specifically as A with T, and C with G to form the characteristic double helix structure of DNA. The structure of viral DNA data, whether linear, circular, single-stranded, or double-stranded, directly impacts how the virus functions and interacts with host cells during infection. The various structural types of viral DNA are illustrated in Fig. 1 [1,4].

**Fig. 2:**
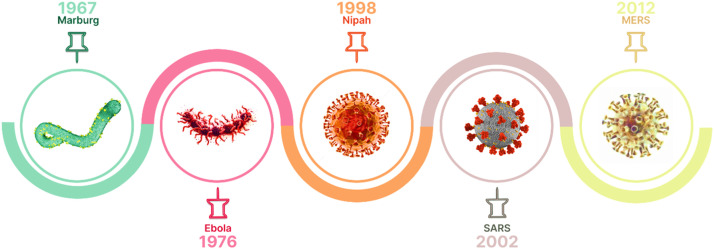
Famous and fetal viruses’ history.

Viruses have posed serious global threats, causing several pandemics with severe consequences. As shown in Fig. 2, among the most fatal viruses, Marburg is one of the most lethal, with a mortality rate of approximately 80 %. The Nipah virus also has a high fatality rate, often exceeding 50 %. Ebola, known for causing severe hemorrhagic fever, has recorded fatality rates ranging from 25 to 90 %, depending on the strain and the capacity of healthcare systems to respond. MERS, a mutant form of coronavirus, has an estimated fatality rate of around 34 %. Lastly, SARS, another coronavirus outbreak, had a lower fatality rate of approximately 10 %, but caused global concern due to its rapid transmission. These viruses highlight the importance of monitoring, preparedness, and rapid response to infectious diseases worldwide [3].

Fig. 2 presents a history of lethal or pandemic viruses and their outbreak dates. By analyzing the virus’s nucleotide sequences, we aim to gain a deeper understanding of genetic structures, identify novel patterns, and uncover key biological markers. As previously mentioned, viruses rely heavily on their DNA for functionality. Genetic sequences are read by dividing them into groups of three nucleotides, known as codons, which constitute the genetic code. Each codon corresponds to a specific amino acid, playing a crucial role in protein synthesis.

In this research, a novel approach based on extracted features from the graph data structure was presented for analyzing the genetic architecture of viral genomic sequences. This approach was tested on various virus types and effectively identified distinguishing features among them. For each case study, a set of features was generated that, according to specific criteria, enabled the differentiation of virus types. The proposed approach was applied to both common and rare viruses, successfully distinguishing each type with high accuracy using graph-derived features. The role of graph algorithm-derived features in the structure of genomic sequences.

In other words, this interdisciplinary research in the field of bioinformatics aims to extract features and classify genetic samples of viruses at the nucleotide level. Its innovations include the application of complex network concepts and graph centrality algorithms in the feature extraction phase. Each genomic sample is represented as a graph, with each codon acting as a node in this directed, weighted graph. Based on the weights of the graph's edges, the final features were extracted using the eigenvector centrality algorithm, as well as in-degree and out-degree metrics. In the classification phase, interpretable decision tree models were used. This approach also established rules regarding codon features that can have biological interpretations. Another significant aspect of the research, from a genetic standpoint, is the model's independent performance across different types of viruses, regardless of their internal structure and length. Details of the proposed approach will be explained further in the following sections.

## 2 Related works

Over the past two decades, the application of computer science algorithms such as text mining, graph analysis, and signal processing algorithms on genetic data has grown significantly. Most of these studies have focused on cancer and other common genetically influenced diseases [[5], [6], [7], [8], [9]]. However, the global outbreak of COVID-19 changed the attention of researchers toward viral diseases, particularly those with widespread impact. In recent years, various computational approaches have been developed for the analysis and classification of viruses at both the nucleotide and protein levels.

Among the early studies that applied graph-based methods to genetic data is [10], which introduced a novel approach using weighted and directed graphs to analyze DNA similarity across 12 mitochondrial DNA sequences from the NCBI database, achieving 100 % accuracy. Additionally, various graph-based works in the field of genomics have been conducted on cancer data, which [10] has reviewed them. Some of these graph-based works [11], has also focused on genetic data in the form of amino acids sequences. Additionally, research on the role of graph metrics in intracellular chemical structures has been previously conducted, although it may have been limited to specific samples [12]. In [13], a graph-based approach is used for analyzing DNA sequences using the k-means clustering algorithm to enhance the understanding of genetic relationships. This research used a method that considers each species as a distinct weighted directed graph. Knowledge graph concepts used in [14] for modeling the genomic sequences by designing a tool.

Several studies have also been carried out in the field of classifying genetic data using various approaches. For example [15] uses the XG Boost model, achieves 100 % accuracy in SARS-CoV-2 classification, 86.9 % for binary and 67.5 % for multi-class, for species and continent classification using 60,063 WGS, computed into 16-dimensional features. Also [16] applies three numerical mapping techniques to convert DNA sequences into numerical data for liver cancer classification using machine learning models (1D-CNN, VGG16 + SVM, fine-tuned VGG16). The fine-tuned VGG16 model achieves 100 % accuracy.

Some of the papers used graph or probabilistic graph modeling, but their dataset is not focused on viral samples. As an instance, [17] used a kernelized classification with machine learning models’ approach by Markovian analysis of dinucleotide patterns to recognize cancer. Their dataset contains 1111 samples from NCBI GenBank. A weighted graph model analyzes DNA similarities in 10 cotton species using K-means clustering and phylogenetic tree construction with 99.56 % accuracy in viral DNA samples classification by random forest-based classifier [18]. The [19] compared several machine learning algorithms such as CNN, KNN, MLP, and SVM for detecting SARS-CoV-2 sequences with NCBI, and GISAID datasets. SVM classifier obtained 99 % accuracy for binary classification and 98 % for multi-class classification.

In signal processing scope, several studies performed on genomic sequences classification. In [20], the authors classify 107,000 coronavirus genomic strings with LPC (Linear Predictive Coding), and SVD (Singular Value Decomposition) feature extraction methods. A SVM model achieves 99 % accuracy. Also, FFT-based algorithms used on [21], which distinguish coronavirus samples from influenza virus by KNN algorithm. Also, KNN algorithm used in [22], which viral genomes are predicted from raw human DNA on 19 metagenomic data. In this research k-mer counting and the bag-of-words technique used as feature extraction algorithms and KNN classifier obtained 98.6 % accuracy.

Research in the field of virus classification based on their genomic sequences can also be examined through the lens of medical science. Some studies have focused on various viruses, while others, such as [23], have been limited to variants of the coronavirus. In this study, the XG Boost algorithm was used for classification, achieving an accuracy of 98 %. In [24], the XG Boost algorithm was also employed and tested on three types of viruses. It was noted that the algorithm's accuracy depends on the length of the studied sequences, with a maximum accuracy of 98 %. The use of ensemble methods based on decision trees has also been observed in other studies, such as [25], which tested multiple coronavirus variants and achieved an accuracy higher than 99 %.

Deep learning algorithms are also used on several papers within this scope. For example, an explainable CNN model is utilized (XCNN-SC) to classify seven SARS-CoV-2 and detect mutations with 99.78 % accuracy with feature extraction, label encoding, and a single-layer 1D-CNN [26]. Some innovative research was done based on the samples geographic location, such as [27]. They classify COVID-19 sequences by MLP, CNN, and Bi-LSTM to analyze the effect of travel restrictions on virus evolution in nine ASEAN nations on the GISAID dataset. The [28], is another deep learning research, which used DNACoder, an attention-based CNN-LSTM, to enhance genomic sequence compression for efficient storage and transmission. Markov Model and One-hot encoding achieved 92.16 % training accuracy.

The basis of some studies was the textual representation of biological sequences for nucleotide characters. For example in [29], six types of viruses were analyzed in the form of nucleotide characters, where the use of CNN and LSTM achieved 93 % accuracy in distinguishing the viruses. The use of the CNN algorithm in other studies, such as [30], has also shown better performance, successfully achieving 96 % accuracy on a much larger dataset. Another relatively recent study in the field of deep learning is [31], which employed the BERT algorithm on >40,000 samples and achieved 97 % accuracy. It should be noted that, classical artificial neural networks such as MLP have also been used in this scope. For instance, in [32], and on 24 types of viruses with an imbalanced distribution, a success rate of 98 % accuracy was achieved.

VirGrapher enhances viral sequence identification by treating full sequences as graphs to preserve relationships. Graph Convolutional Networks (GCNs) and Graph Self-Attention (GSA) were used with 85.90 % in human gut metagenomes [33]. Hybrid deep learning models for COVID-19 genome classification by CNN-BLSTM-Att is used which achieved 99.99 % accuracy. Methods that act as prosperous ones are k-mer encoding, Count vectorizer, and the sliding window methods [34]. TransGINmer enhances virus identification in metagenomes, outperforming existing methods which tested on 15,037 viral genomes with 96.28 % accuracy using k-mer frequency embedding and multi-head self-attention for precise classification [35].

In [36], natural language processing methods have been used to classify untransformed genetic data. Transformer-based algorithm has the main role of this paper, which also used in [37]. In [38], a transformer-based approach was utilized for the three-class classification of coronaviruses. Another version of transformers was tested on a different virus (influenza) in [39]. Indeed, the use of transformers has had diverse applications in the field of genomics and has also been reported in the detection of specific regions or types of genetic data [40].

Additionally, the idea of presenting a graph-based feature extraction approach in [41], which is based on Markov graph centrality, can also be mentioned. In this research, the decision tree achieved over 99 % accuracy in enabling multi-class differentiation of the samples. Other research is also being conducted in the field of genetic data using machine learning algorithms to address issues in this area [42].

Despite the extensive volume and diversity of research conducted in this field, there remain various ambiguities and questions regarding genetic data. High-accuracy classification of different genetic samples may be one of the primary objectives of these inquiries. Providing interpretable models and linking them to genetic concepts is another goal that could open new perspectives in this area. Furthermore, the role of developing algorithms in computer science and signal processing within interdisciplinary fields such as bioinformatics should not be underestimated.

## 3 Methodology

**Fig. 3:**
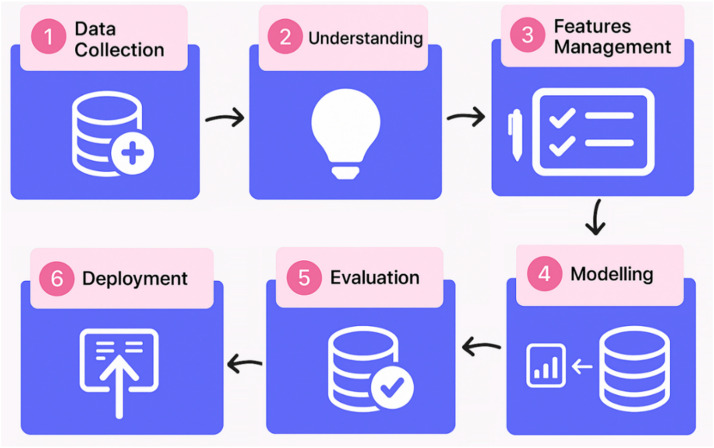
Data mining process of research.

The overall process and flowchart of this research follow a typical data mining structure, with stages aligned to the standard steps of a pattern recognition model. This process is illustrated in Fig. 3.

As illustrated in Fig. 3, the process of this project is structured such that the dataset is first collected, followed by a thorough understanding of the samples and their characteristics. Unlike many previous studies that relied on text mining or signal processing techniques, this research adopts a graph-based approach. Feature extraction and management are also carried out based on this graph-oriented framework. In the modeling phase, supervised machine learning algorithms are employed to perform classification using the extracted features. A decision tree classifier is specifically used to ensure interpretability and to produce meaningful, explainable results. The evaluation will be based on common machine learning metrics. Furthermore, additional experiments were designed to demonstrate that the effectiveness of the proposed approach is not limited to a specific virus.

### 3.1 Dataset

This dataset was collected from the virus section of the NCBI GenBank database [43] and provides various types of viruses, detailing their biological and structural characteristics. There are multiple classifications and taxonomies based on various criteria (such as cellular structure, genome type, shape, realm, family, transmission rate, and safety level). These factors were taken into account to assess the performance independently of type, structure, and length of the proposed approach. Additionally, viruses with high infection rates or widespread prevalence, such as COVID-19 and influenza, were prioritized. On the other hand, similar viruses in terms of clinical symptoms were also selected. The accompanying Table 1 categorizes these samples to facilitate comparison and analysis, making the dataset valuable for virology research, academic studies, and bioinformatics applications. It should be noted that series of these samples previously used in [20,21,41] studies.

**Table 1:** Dataset details.

**#**	**Virus**	**Count**	**Min**	**Max**
1	**Coronavirus**	65,060	27,121	30,255
2	**Influenza**	53,176	1408	2658
3	**Norwalk**	2687	6525	7778
4	**Coxsackie**	1410	7055	7621
5	**Crimean-Congo**	145	11,838	12,228
6	**Cytomegalo**	312	233,028	243,962
7	**Ebola**	2186	17,554	18,996
8	**Encephalitis**	133	170,059	177,317
9	**Hepatitis B**	8388	2729	4224
10	**Hepatitis C**	794	8047	9591
11	**Varicella**	72	123,769	125,305
12	**HIV**	18,357	8500	15,524
13	**Lassa**	263	11,766	11,928
14	**Marburg**	58	18,997	19,115
15	**Mastadeno**	1392	33,409	36,571
16	**Measles**	575	14,010	15,900
17	**Metapneumo**	356	13,049	13,516
18	**Mumps**	550	14,048	15,385
19	**Papillomavirus**	1136	6350	6562
20	**Parainfluenza**	34	7155	8182
21	**Poliovirus**	723	15,246	17,384
22	**Pox Monkey**	612	7019	7500
23	**Respirovirus**	468	195,410	208,626
24	**Rhinovirus**	1559	14,134	19,257
25	**Rotavirus**	1966	6805	7304
26	**RSV**	1836	3006	3308
27	**Rubella**	107	14,206	15,322
28	**Zika**	603	9726	9777
29	**Yellow fever**	207	10,076	11,155
30	**Dengue**	8063	10,004	11,195
	**Total**	173,228	1408	243,962

Table 1 provides more details about the selected data and their information in various columns. It includes the names of the viruses, statistical information regarding the number of selected samples from each type of virus, minimum, and maximum length. As is evident, an equal number of each virus was not selected. This was due to the unavailability of uniform data for all viruses. However, for common viruses like corona and influenza, a large number of samples were chosen. Also, various variants of these viruses used to better assess the independence of the proposed approach concerning this aspect and its generalizability.

The data was selected in such a way that for a specific type of virus, there is minimal variation in length. In other words, the standard deviation of the sequence length values within each viral category is low and close to the reference genome length or its common genomes. This is important because for each virus, there are also samples with significantly shorter lengths included in this dataset, which represent partial sequences or specific genes of that virus. Also, this research did not focus solely on one specific type; From a genomic shape perspective, not all selected viruses were linear; viruses like Papillomavirus, Lassa, and Rotavirus are circular or other modes. Furthermore, in terms of viral taxonomy, not all viral diseases were categorized under a specific realm. Most viruses belong to the Riboviria type, but others such as Varicella and Cytomegalovirus fall under the Duplodnaviria type, and Mastadenovirus belongs to Varidnaviria type.

One of the stages of the research was dedicated to data preprocessing. Given that the proposed approach was designed at the nucleotide scale, the data needed to be examined accordingly. To this end, data containing ambiguous characters (other than A/C/G/T) were identified and cleaned. One of the key tasks performed prior to the feature extraction phase was the graph-based modeling of the raw nucleotide sequences. For this purpose, each sequence was treated as a string of text, from which overlapping substrings of three characters in length were extracted using an approach similar to the 3-mer method.

One of the main preprocessing phases of the research involved the normalization of values. Given the varying lengths of the samples, a technique was needed to ensure the model's performance was independent of sample length. Therefore, the number of observations for each node following any specific node was taken into account. In other words, a 64×64 matrix was created for each sample, recording the number of observations for each of the 64 possible nodes following a particular node. Normalization was then performed based on the sum of the values in each row of the matrix. The probability of observing each node after a specified node became dependent on all possible states. This operation ensured that the values in the matrix entries for each sample fell within the range of 0 to 1. Consequently, this approach made the algorithm's process independent of the lengths of the samples.

### 3.2 Feature extraction by graph algorithms

Graph algorithms are made up of graph data structure elements, especially nodes (or vertices) and edge (or links). In this study, a directed and weighted graph structure is employed on each genomic sequence sample. In this study, each nucleotide sequence was modeled as a graph by representing nucleotide triplets as nodes. Specifically, every three-nucleotide segment corresponds to a single node within the graph. To incorporate the directed and weighted nature of the graph, the frequency with which one triplet follows another in the sequence was calculated. This frequency was then normalized to derive the probability of observing a given triplet immediately after another, which served as the weight of the directed edge connecting the corresponding nodes [44].

**Fig. 4:**
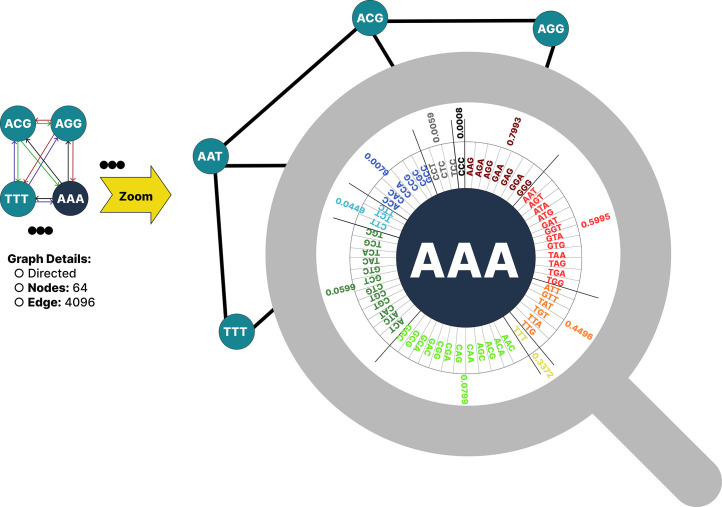
An overview of the graph focusing on one of the nodes and its related edges.

In this research, for each DNA sample, a graph with 64 nodes is constructed, where each node represents a trinucleotide sequence. The graph is represented as a 64×64 matrix that encodes the weighted connections among the trinucleotides. Then, centrality measures are calculated for each node, generating 64 distinct features per sample. Although visualizing the graph is impractical due to the large number of nodes, an example illustrating the outgoing edges from a sample node is presented in Fig. 4. As shown in the figure, each node is connected to 64 nodes, but the weight of their connecting edges varies. The figure zooms in on node AAA and the 64 edges connected to it as an example.

As shown in Fig. 4, the graph consists of many nodes and edges (64 nodes and 64 edges for each node). Generalizing this to the entire graph, it would form a complete graph with 64 nodes connected by all possible edges between node pairs. The primary goal of this graph-based modeling of genomic sequences is to analyze each sequence individually as a graph, according to the virus type or label it represents. In graph analysis, certain metrics are node-dependent and quantify the strength or influence of nodes within the network. Other metrics evaluate the flow of information throughout the graph, assessing how much information nodes send and receive.

Centrality measures are powerful tools for identifying major nodes within sophisticated networks. In directed networks, node has three kinds of degree centrality: in-degree (edges pointing toward a node), out-degree (tracks edges originating from a node) and total-degree (sum of in-degree and out-degree). When a node has high rate of connections which means this node due to having a strategic position in the network is called more centrality node. While total connections are few, in other words, outgoing and incoming connections are few which means this node is not as important as the other nodes [45].

**Fig. 5:**
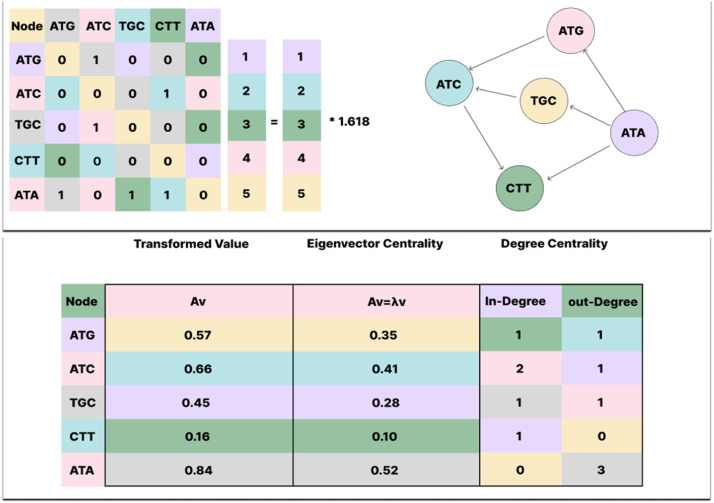
Graph centrality & corresponding matrix eigenvector calculation example.

Eigenvectors are specific vectors that keep their direction while a linear transformation is applied. These are non-zero vectors, when multiplied by a matrix, lead to a scaled version of themselves. Eigenvectors can be separated into right eigenvectors, multiplied on the right, and left eigenvector multiplied on the left. The basic equation for eigenvectors is Av=λv, where A is the matrix, v is equal to the eigenvector, and λ represents the eigenvalue. The finding eigenvectors process includes two primary steps. Firstly, find eigenvalues by solving det (A – λI) = 0. Secondly, for per eigenvalue λ, find the corresponding eigenvector by solving (A – λI) *v* = 0 which demonstrates the eigenvalue equality, where I in formula is the identity matrix. Negative eigenvalue results in a direction during the transformation [46]. Matrix eigenvector transformation is illustrated in Fig. 5.

An important issue that arises after forming the graph is the selection of an appropriate metric for valuing or weighting the nodes. Centrality measures possess different meanings, and in this context, the influence of a node may not be the only critical aspect. The interactions between individual nodes can also be significant. Consequently, three graph metrics, which carry different concepts, have been utilized. In this research, three types of centrality metrics were used to calculate graph centrality, which are summarized in Table 2. This table includes the name of the metric, a general description, and its mathematical formula. It should be noted that the graph considers as G = (V, E) and $A\in R^{64*64}$ as its adjacency matrix.

**Table 2:** Centrality metrics information.

	**Name**	**Description**	**Formula**
1	In-Degree	How much a node receives from other nodes, useful for measuring popularity or authority	$C_{In}\left(i\right)=\;\sum_{j=1}^{n}A_{ji}$
2	Out-Degree	How much a node sends to other nodes, often used to measure activity or influence	$C_{Out}\left(i\right)=\;\sum_{j=1}^{n}A_{ij}$
3	Eigen-Vector	Quality of connections by captures global influence propagation through the graph, useful for capturing global influence within a network	$C_{EV}\left(i\right)=\;\frac{1}{\lambda }\sum_{j=1}^{n}A_{ji}C_{EV}\left(j\right)$

Out-degree centrality identifies features that cause or influence others strongly. In-degree centrality identifies features that are influenced by others. Eigenvector centrality identifies features that are globally important in the network of interactions. In Eq. (1), a hybrid formula derived from the three mentioned metrics.

(1) $$C_{hybrid}=C_{In}\left(i\right)*C_{Out}\left(i\right)*C_{EV}\left(i\right)$$

This metric simultaneously possesses the properties of all three centralities in terms of receptivity, dissemination, and overall connectivity of the network. The output is a numerical value, where a higher number indicates the significance of that feature in both local and global scales of the network. However, each of the metrics was also tested individually in this research, yielding different rankings for each node. A node with a high in-degree is regulated by many others and may serve as a key target or a central response hub within the network. Also, A node with high out-degree is a master regulator, possibly a transcription factor that affects many downstream processes. Eigenvector centrality is a global measure that not only considers the number of a node’s connections but also the importance of the nodes to which it is connected. This metric identifies nodes that are linked to other influential nodes.

The combined use of these three centrality measures can prioritize nodes that are both structurally central and functionally influential. These nodes may serve as critical control points within the system and could be of particular interest in genomic or regulatory network analysis. Eigenvector centrality is a metric that assesses the importance of each node based on the number and quality of its connected edges. This metric helps identify nodes that are targets or influences of other nodes. Out-degree centrality has a similar definition and is used to identify nodes that can exert greater influence over other nodes.

Overall, in-degree and out-degree centrality metrics are crucial for identifying information flow. However, using each centrality metric as an independent feature enhances the interpretability of the model. However, instead of having 64 features, we will have 192 features per instance, which increases computational complexity. In this context, the centrality value of a node or its rank among 64 cases may not be particularly significant. Instead, the values obtained for each sample are used as threshold limits for each type of virus in their respective classifier models.

### 3.3 Classification by decision tree algorithm

In the final phase of the study, a data mining model based on machine learning algorithms was employed for evaluation. Since the dataset consisted of labeled samples from various virus types, the problem was framed as a supervised classification task. In the domain of supervised learning, numerous algorithms are available, each with its own strengths and limitations. This research prioritized a white-box approach by utilizing an interpretable model during the classification phase. Nevertheless, other algorithms were also tested in selected experiments to compare performance.

**Fig. 6:**
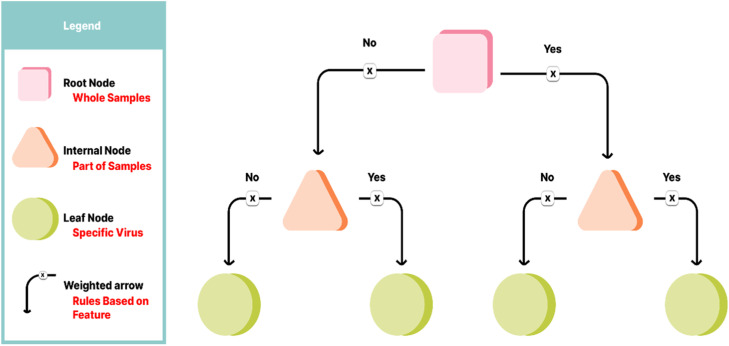
Decision tree elements.

A decision tree is a visual representation used to make decisions and anticipate consequences. It imitates human decision-making via separating data into branches based on feature values. Each node demonstrates one trait or condition and nodes based on traits will be divided. This algorithm consists of a root node represents the entire dataset and is the start point of the tree, which branches that arrows links nodes and demonstrate possible decisions due to result of conditions in previous node, internal nodes which represent decision relied on traits, eventually, leaf nodes which are renowned as final result [47]. The elements of a decision tree are demonstrated in Fig. 6.

The decision tree algorithm begins at the root node, which poses a question based on a particular feature of the dataset. According to the value of that feature, the dataset is divided into branches, and the decision path is determined by evaluating conditions at each node. This branching continues recursively through internal nodes until no further conditions remain to be evaluated. The process terminates at leaf nodes, which provide the final output. In classification trees, the output is a class label, whereas in regression trees, it is a continuous value. In this research, a classification tree is used to assign virus types based on extracted features.

It should be noted that, other classification algorithms such as neural networks, SVM, KNN, and logistic regression were also used for comparison. However, one of the objectives of this research was to examine the role of the extracted and selected features in the performance of the classifier. To this end, a decision tree was employed, which is a white-box approach with high interpretability. This approach not only provided better interpretability compared to neural networks and SVM but also had a faster processing speed. Additionally, it did not face the statistical distribution constraints, nonlinear space limitations, and specific techniques for managing imbalanced data that logistic regression does. Compared to other interpretable classifier methods, decision trees have their own advantages, including the ability to rule visualization, reduce nonlinear feature space limitations, and offer computational and statistical simplicity.

### 3.4 Performance evaluation

The performance of the proposed algorithm is evaluated using common metrics such as accuracy, precision, recall, and F1-score, which collectively assess the quality of predictions. Additionally, confusion matrices are employed to provide insights into true and false classifications, while ROC curves are used to analyze the trade-off between sensitivity and specificity. To ensure consistent and reliable results, cross-validation is applied, which helps prevent both overfitting and underfitting [47].

**Fig. 7:**
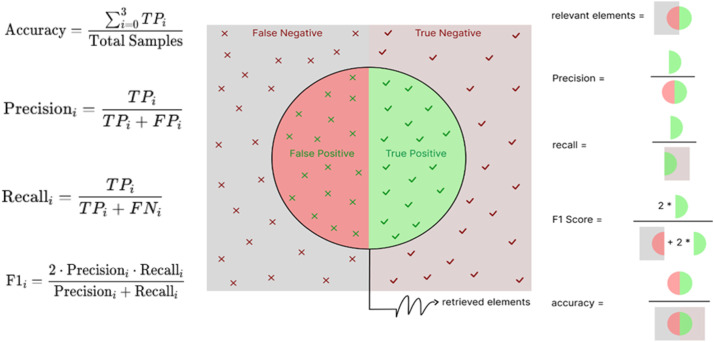
Performance evaluation metrics visualization.

In this study, 5-fold cross-validation is used for model evaluation. The dataset is divided into 5 equal parts (folds); in each iteration, the model is trained on 4 folds and tested on the remaining one. This process is repeated 5 times, with each fold serving as the test set exactly once. The average performance across all iterations provides a robust and unbiased estimate of the model’s effectiveness. Applying this method ensures a more reliable evaluation of the decision tree classifier by utilizing different subsets of the data for training and testing [47]. In machine learning evaluation step, several metrics can be used based on true positives (TP), true negatives (TN), false positives (FP), and false negatives (FN) concepts. The formulas for accuracy, precision, recall, and F1-score are presented in Fig. 7.

As illustrated in Fig. 7, the evaluation of a classification model is based on the count of correct and incorrect predictions by using accuracy and F1-score. F1-score is a crucial metric that combines precision and recall metrics to provide a balanced assessment of a model’s performance. Unlike simple accuracy, which only reflects the proportion of correct predictions over the entire dataset, the F1-score offers a more nuanced view by considering both the model’s ability to correctly identify positive instances and its capacity to retrieve all relevant positive cases. This metric is particularly valuable in scenarios with imbalanced datasets, where relying solely on accuracy may lead to misleading conclusions. The F1-score helps to ensure a fair evaluation by emphasizing the trade-off between precision and recall. While the figure depicts a binary classification scenario, in multi-class classification, the confusion matrix extends to reflect the correct predictions for each class.

## 4 Results

The implementation of the proposed approach was carried out using MATLAB 2018 software, and the results are detailed in the following subsections.

### 4.1 Feature importance analysis results

**Fig. 8:**
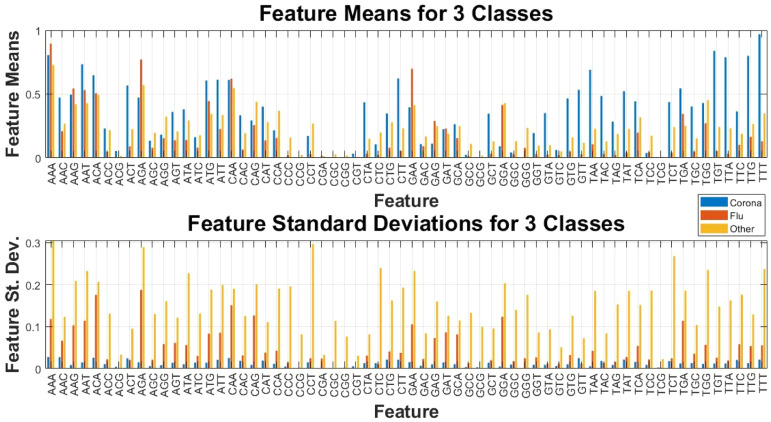
Extracted features means and standard deviation values per each kind of virus.

This research was conducted on several datasets collected from the NCBI website, addressing the topic from various perspectives. The proposed algorithms were developed for feature extraction and the creation of rules from each case study extracted 64 features related to the eigenvector centrality of each case study. The aim of this experiment is not to find the maximum or minimum value among these 64 values, but rather to identify values for the features that enable the differentiation of each type of virus. Fig. 8 illustrates the graph-derived features from the three values: eigenvector, in-degree, and out-degree centrality.

**Fig. 9:**
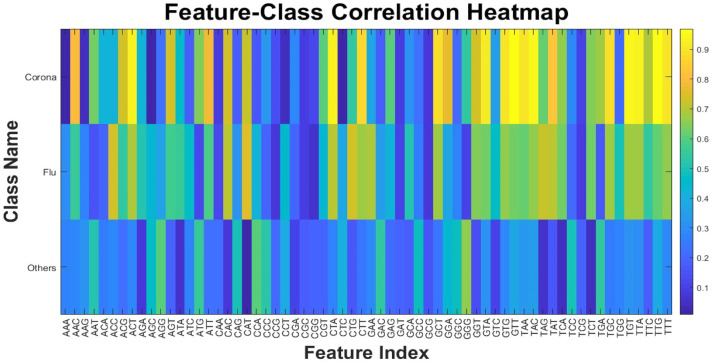
Correlation heatmap of extracted features with 3-class labels.

Fig. 8 presents the final features of the research, which serve as the basis for the subsequent phases of the study, specifically the classification phase. In this figure, the mean and standard deviation for all three types is highlighted by different colors. It is evident that the standard deviation of the samples of other viruses is significantly higher, which is expected since this class includes 28 types of viruses, and the diversity of their results is reflected in this statistical component. One of the aims of this research is identifying features (minimum features) among these values for the classification and differentiation of virus types based on binary, or multi-class definitions. It should be noted that to rely solely on the mean or standard deviation of the feature values; additional statistical tests have also been conducted on the feature space of the classes. Among these, the correlation of all features with the assigned labels is illustrated in Fig. 9.

In Fig. 9, the correlation values of each feature with their specific three-class labels are displayed as a heatmap image and three separate charts. The blue color is related to the lower correlation, while yellow indicates higher values. Each of the three sections can be analyzed individually, but what matters for the classification phase is identifying features that provide the best separation boundary or highest evaluation metric for distinguishing the three classes. Further details on the feature selection approach and classification according to the number of class types will be provided in the following subsections.

### 4.2 Binary-class results

In the initial experiment, the coronavirus samples (from various variants) were compared with the influenza and other viruses. Thus, a binary-class classification problem was defined, and various classification algorithms were tested to analyze the feature space. In this experiment, 65,060 samples of the coronavirus from new variants (such as Alpha, Delta, and Omicron) and previous versions (such as MERS and SARS) were analyzed, along with 108,168 samples of other virus types (such as influenza). The goal of this test is to design an interpretable model that allows the examination of the features extracted by the rule-based algorithm developed. Therefore, the decision tree algorithm is used, which requires significantly less time and memory compared to other classic machine learning algorithms such as KNN, SVM, and artificial neural networks.

When designing a machine learning model, one of the key influencing parameters is the number of selected features for classification. About the utilized decision tree in this experiment, a set of common and uniform parameters was employed. The split criterion used was Gini Index (GDI), and each node was configured to have a maximum of two children. However, several parameters were adjusted based on the number of samples, the number of classes, and other influencing factors during the training and testing phases.

**Fig. 10:**
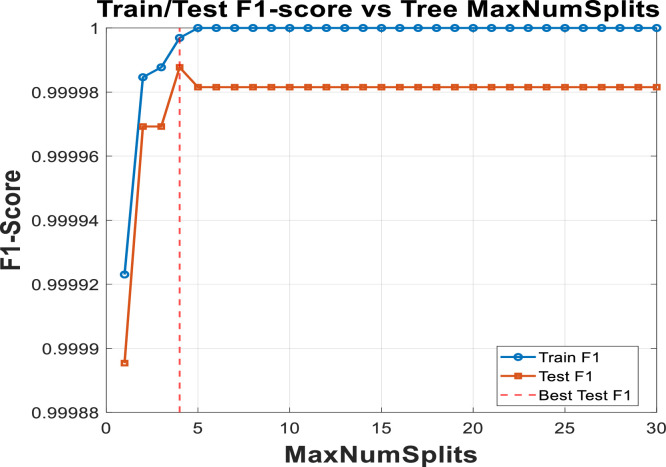
Maximum number of splits efficiency on F-1 score values of binary-class classification problem.

In decision trees, one of the important parameters of features is controlled by the maximum number of splits, which directly relates to the number of leaves in the resulting tree. This parameter directly corresponds to the number of extracted rules. For this purpose, the collected data were trained and tested using a decision tree algorithm with a 5-fold cross-validation approach. In this experiment, the maximum number of splits parameter in the decision tree was treated as a variable, and its impact on accuracy and F1 score was evaluated by incrementally increasing it one by one. The results are illustrated in Fig. 10, where the horizontal axis represents the variable parameter and the vertical axis shows the obtained F1 score.

**Fig. 11:**
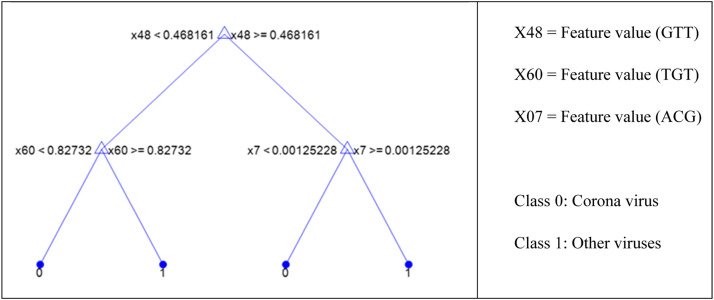
Extracted rules and a decision tree with 4 splits or leaves.

Fig. 10 illustrates the impact of changing the number of splits in the decision tree from 1 to 30 on the F1 score metric using 5-fold cross-validation. The changes in the training and testing phases are shown in two separate colors. In both the training and testing phases, the stratified k-fold validation technique was used to address the imbalanced mode of data partitioning. By examining the variations in the training and testing graphs and the gap between them, it can be inferred that having 4 or 5 leaves in the tree represents the optimal scenario. If classification is performed using 4 leaves, which correspond to the centrality values of the nodes in this study, 4 rules will also be extracted, as depicted in Fig. 11. This decision tree achieved an accuracy of over 0.99 and an F1 score exceeding 0.99, enabling the effective differentiation and identification of coronavirus samples from other viruses.

As shown in Fig. 11, for each of the two classes (0 or 1), two rules have been extracted, with the class type indicated by 0 and 1 at the leaves of the tree. Class 1 corresponds to coronavirus samples, while class 0 pertains to other viruses. The terms X7, 48, and 60 in the image refer to the values of the features extracted from the graph nodes. According to the previous figures, feature 7 corresponds to the ACG node centrality-based feature, feature 60 to the TGT, and feature 48 to the GTT node centrality-based feature. In other words, assuming that the feature defined for each node is equal to the product of the eigenvector centrality and the input and output degree centrality values, four rules can be extracted from this tree. For each class, two rules have been extracted, which are presented in Table 3 with the operator "OR" between the rules. The value of each feature is also indicated by the "feature" term.

**Table 3:** Extracted rules from the decision tree with 4 features on binary-class data.

	**IF …**	**Class**
1	(feature_GTT < 0.468161) AND (feature_TGT ≤0.82732) **OR** (feature_GTT ≥ 0.468161) AND (feature_ACG ≤0.00125228)	Corona
2	(feature_GTT < 0.468161) AND (feature_TGT ≤0.82732) **OR** (feature_GTT ≥ 0.468161) AND (feature_ACG ≤0.00125228)	Non corona

**Fig. 12:**
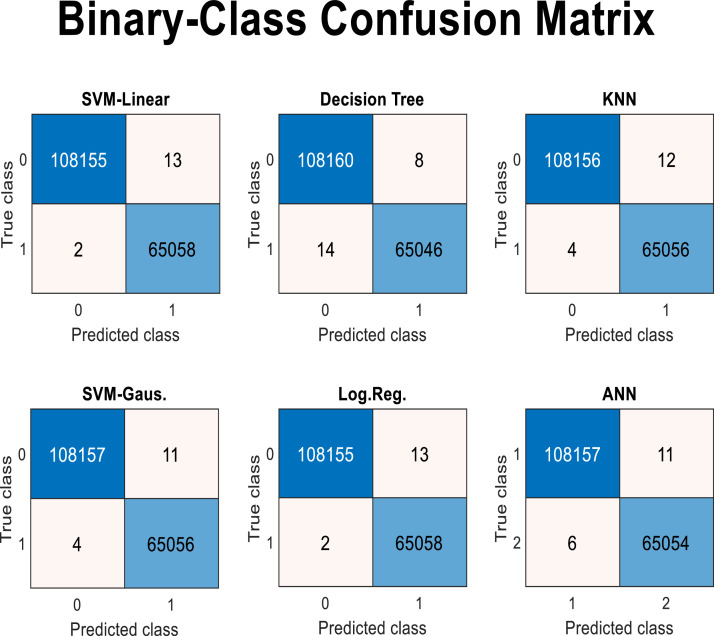
Confusion matrix of several algorithms on a binary-class classification problem.

As shown in Table 3 and Fig. 11, the role of feature number 48, which corresponds to the value of the “GTT” node feature, is significantly more pronounced. Even with this single feature, the class type can be determined with high accuracy and F1 score (above 0.99). If it’s (the GTT node) value exceeds 0.461945, it belongs to the coronavirus class; otherwise, it is classified as another type of virus. Therefore, if only a specific feature value (the GTT node) is used for classifying samples, it demonstrates a high ability to distinguish coronavirus samples from other viruses. This finding is not solely attributed to the decision tree classifier; experiments conducted with other classifier algorithms have confirmed the same outcome. Fig. 12 presents the results of this experiment by 5-fold cross validation using linear SVM (support vector machine), Gaussian SVM, ANN (artificial neural networks), KNN (K-nearest neighbors), and logistic regression, along with the resulting confusion matrix.

As shown in Fig. 12, all machine learning algorithms, yielded very good results in classifying the two classes. In all the matrices of this figure, the first row refers to non-coronavirus samples, while the second row pertains to coronavirus samples. The false positive and false negative rates for all algorithms are quite low. Although the results from the decision tree algorithm appear to be worse than those of the other algorithms, the lower computational time and fewer parameters required by this algorithm are significant advantages. Additionally, it is important not to overlook the lower interpretability of other algorithms compared to the decision tree.

**Fig. 13:**
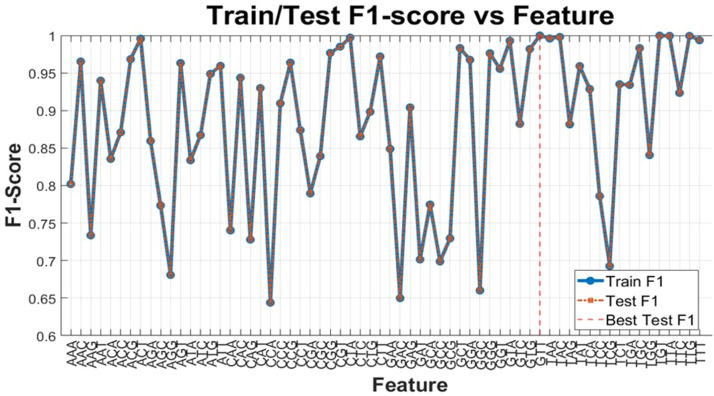
F-1 score of the decision tree algorithm with all features.

Another experiment that can be conducted based on this topic is to examine the role of the values obtained for other nodes in the graph. In other words, the question of this experiment was whether considering other features as the sole influential feature for the classifier would still yield high accuracy and F1 scores. To this end, an experiment was conducted using 5-fold cross-validation, and the impact of individual features on the resulting F1 score and accuracy was analyzed, as illustrated in Fig. 13. In this figure, both the F1 score results from the training phase and the testing phase are presented, with the difference in results being similar to that in previous figures, at approximately 10 to the power of negative five.

In Fig. 13, the horizontal axis represents the names of the features, and the F-1 score indicates the performance achieved by considering only that feature for training and testing the decision tree. In fact, this experiment demonstrates how well samples from the two classes can be separated using features extracted from the triplet codons. The results show that the extracted features provided a discrimination power for the samples with an accuracy ranging from 0.63 to 0.99. Among these features, “GTT”, “ACT”, “CTA”, “GTA”, “TTG”, “TTA”, “TGT”, “TAC” and “TAA”, yielded very good results, operating close to 100 %. Examining the similarities of these features from a genetic perspective may reveal additional characteristics. For instance, one can consider the proteins resulting from the translation of these triplet codons, where GTT and GTA translate to a specific protein, while TTG, TTA, and CTA also correspond to another specific protein.

As indicated in Fig. 10, Fig. 13, even with just one feature, the testing phase yields high results from the outset. If we aim for computational simplicity in the learning phase while maintaining explainability, we can also assess the impact of a single feature on other evaluation metrics. In other words, extracted features are highly informative. The model achieves high generalization without much overfitting. These results also show a significant relation with the correlation of each feature against the assigned label. It is possible that the high values of each feature can be attributed to the strong correlation between all pairs of features, as the pairwise correlation among the triplet codons is defined based on the concept of features outlined above. This concept of features is defined according to the in-degree and out-degree of each node.

These results are hypotheses derived from the tests conducted on this dataset, and similar tests can be performed on other datasets as well. However, it should not be forgotten that these experiments were aimed at distinguishing the coronavirus from other viruses, and if another virus is considered, the results will certainly differ. Nevertheless, the feature extraction approach and the interpretable classifier used provide the capability to analyze and draw conclusions from any dataset under various conditions. For other machine learning algorithms, similar approaches can be applied, such as sequential feature selection. Alternatively, filter-based feature selection methods, like evaluating the correlation between features and sample labels, can be used.

### 4.3 Multi-class results

**Fig. 14:**
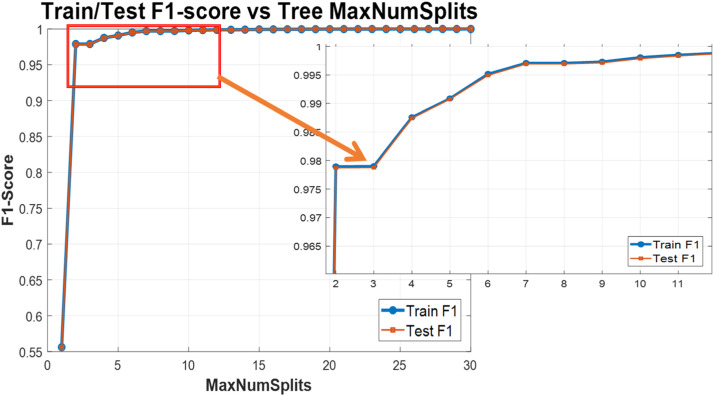
Maximum number of splits efficiency on F-1 score values of 3-class classification.

In this section, experiments were conducted based on three classes: coronavirus, influenza, and other viruses, with labels 1, 2, and 3 assigned to each of them, respectively. For the evaluation of multi-class models, accuracy and F-1 score metrics can be used, starting with experiments using the decision tree algorithm. To this end, the impact of the number of leaves (maximum number of splits) was examined on the F-1 score. The results of this experiment are illustrated in Fig. 14. Again, the results are not limited to the testing phase; and the training phase is also included to address data issues (such as overfitting).

As shown in Fig. 14, the training and testing phases results are very close to each other, with minimal differences between the two phases. Additionally, the differences among the various folds in the 5-fold cross-validation exhibit low variance. This figure consists of two parts: the overall view is shown on the left, while a zoomed-in section focuses on the number of splits on the right side. It is evident that starting with one split does not allow for covering all classes in a three-class problem with a binary tree, resulting in low initial accuracy. As the number of features increases, the F-1 score also rises, but it stabilizes around seven features. However, the continuation of the graph indicates that the maximum score is reached with 25–27 features.

**Fig. 15:**
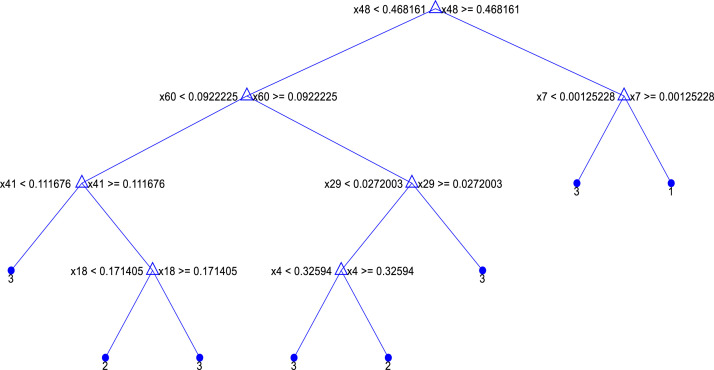
Extracted decision tree with 7 splits or leaves.

If we want to construct a decision tree with lower complexity, using seven features may be sufficient. Tests with a higher number of features, such as 65, were also conducted, but it is essential to consider issues related to machine learning models, such as overfitting, which is more likely to occur in decision trees. Therefore, the tree resulting from a maximum of 7 splits is illustrated in Fig. 15. This figure depicts the tree based on the feature numbers. From top to bottom, the tree utilizes the following features: 48 (GTT), 60 (TGT), 7 (ACG), 29 (CTA), 41 (GGA), 4 (AAT), and 18 (CAC). The leaves of the tree correspond to three types: 1, 2, and 3 classes, which are assigned to samples of coronavirus, influenza, and others, respectively.

In analyzing the tree of Fig. 15, the importance of the GTT feature can again be emphasized, as it plays a pivotal role in distinguishing between the classes. This feature is also utilized at lower levels when using a deeper tree with a greater number of leaves for further sample differentiation. From this tree, eight rules can be extracted to determine the class type of the samples. To this end, Table 3 presents a list of the extracted rules based on the values of the aforementioned features.

As indicated in Table 4, a series of rules has been extracted for each type of virus. Just one rule corresponds to the coronavirus class, which is represented by two general rules. The highest number of rules corresponds to other viruses, which is a natural result given their higher diversity. These rules successfully distinguished samples from the three different classes with 0.99 accuracy and F-1 scores exceeding 0.995. Based on these rules, further analyses can be conducted regarding the triplet codons and their corresponding proteins. Additionally, these seven features can also be observed among the top ten correlated features with class labels.

**Table 4:** Extracted rules from the decision tree with 7 features on 3-class data.

	**IF …**	**Class**
1	(feature_GTT ≥ 0.468161) AND (feature_ACG ≥0.00125228)	Corona
2	(feature_GTT < 0.468161) AND (feature_TGT < 0.092225) AND (feature_GGA ≥ 0.111676) AND (feature_CAC < 0.171405) **OR** (feature_GTT < 0.468161) AND (feature_TGT ≥ 0.092225) AND (feature_CTA < 0.0272003) AND (feature_AAT ≥ 0.32594)	Flu
3	(feature_GTT ≥ 0.468161) AND (feature_ACG < 0.00125228) **OR** (feature_GTT < 0.468161) AND (feature_TGT < 0.092225) AND (feature_GGA < 0.111676) **OR** (feature_GTT < 0.468161) AND (feature_TGT ≥0.092225) AND (feature_CTA ≥ 0.0272003) **OR** (feature_GTT < 0.468161) AND (feature_TGT < 0.092225) AND (feature_GGA ≥ 0.111676) AND (feature_CAC ≥ 0.171405) **OR** (feature_GTT < 0.468161) AND (feature_TGT ≥ 0.092225) AND (feature_CTA < 0.0272003) AND (feature_AAT < 0.32594)	Others

**Fig. 16:**
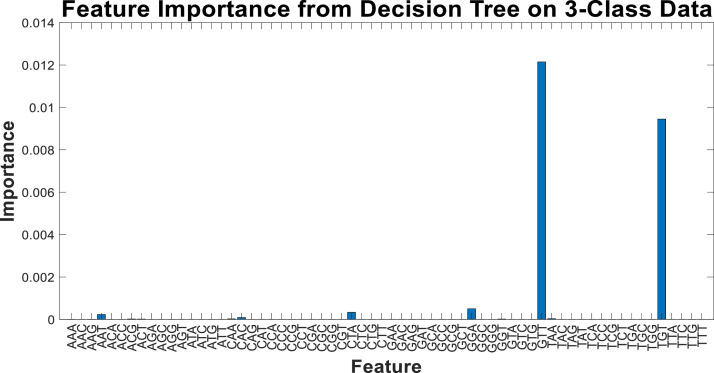
Feature importance of each feature (node).

Another experiment can be conducted with a larger number of features to compare the value of each extracted feature relative to the others. In this regard, Fig. 16 lists the results of the importance of various features, with the feature names displayed on the horizontal axis and their respective values and scores on the vertical axis.

**Fig. 17:**
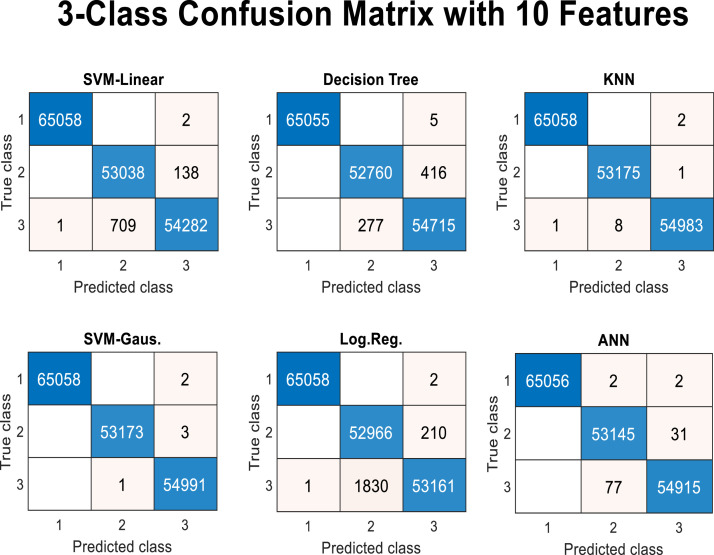
Confusion matrix of several algorithms on 3-class classification problem.

As shown in Fig. 16, about 5 features have more impact out of the 64 features. However, some features also demonstrate their role in the final tree with a higher number of features or splits. It should be noted that a large number of features, while increasing the accuracy of the machine learning model, poses a risk of overfitting. Nevertheless, among these features, the roles of some features, such as two-class and three-class correlation tests, become more evident. Considering this, experiments can be conducted with other classifiers based on the 10 top correlated features with class labels. For the feature extraction phase, the proposed approach was tested using other classifier algorithms, and the confusion matrices of them are illustrated in Fig. 17.

In Fig. 17, the matrices are labeled with three classes of coronavirus, influenza, and other viruses, respectively marked as 1, 2, and 3 in the rows and columns of the matrices. The predicted number for each category by class is also specified in these matrices. It can still be said that the best possible results were not achieved with the decision tree, and the results of non-linear SVM algorithm (Gaussian kernel) or KNN are better. However, the performance of the decision tree also has a very low error, and this low error across all algorithms indicates the effectiveness of the feature extraction and selection algorithm. Of course, other feature selection algorithms (based on the filter, wrapper, or combined type) may also yield better results.

Another multi-class experiment conducted on the collected data relates to the classification of all viruses. In this experiment, each type of virus was considered a separate class, and a 30-class problem was defined, which was tested using a decision tree. To determine the number of leaves or influential features in this tree, similar experiments starting from a maximum of 30 splits were conducted. As the number of classes increases, more details must be considered, and it may be essential to identify influential features in order to calculate separate centralities for each graph node. In other words, for each graph node, three types of features are extracted, and the impacts of eigenvector centrality, in-degree centrality, and out-degree centrality are taken into account separately.

However, it should be noted that in this problem, the statistical distribution of the data was more unbalanced. Therefore, the F1 score criterion and the weighting of the learning and validation models were also considered. Another challenge of this experiment with the decision tree algorithm is to examine and ensure that all types of viruses are present in the leaves of the generated tree. This is important because a specific class type may be repeated more frequently in the leaves of the tree, which is more likely in highly imbalanced datasets.

**Fig. 18:**
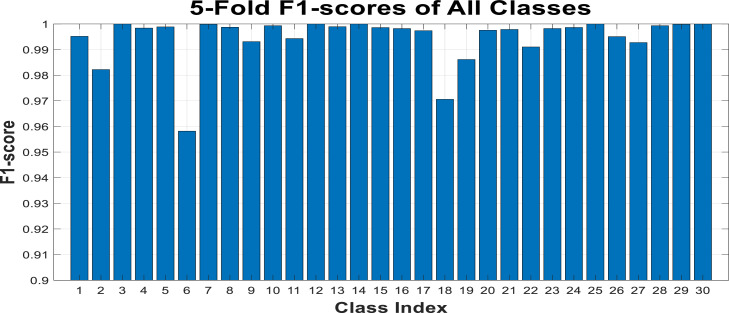
Obtained f-1 score of each class on 30-class problem.

The designed decision tree, with approximately 70 leaves (the maximum number of splits), achieved an F1 score of 0.9918 and an accuracy of 0.9988. To calculate the F1 score for each class, true positive, false positive and false negative rates of each class calculated separately. Then compute precision and recall for each class based on true positives, false negatives, and false positive rates. Based on these, the F1 score for each class can also be calculated. The results are depicted in the form of F1 scores for each class or virus in Fig. 18. The horizontal axis of this figure represents the class or virus number, as set in Table 1, and the vertical axis shows the resulting F1 scores.

**Fig. 19:**
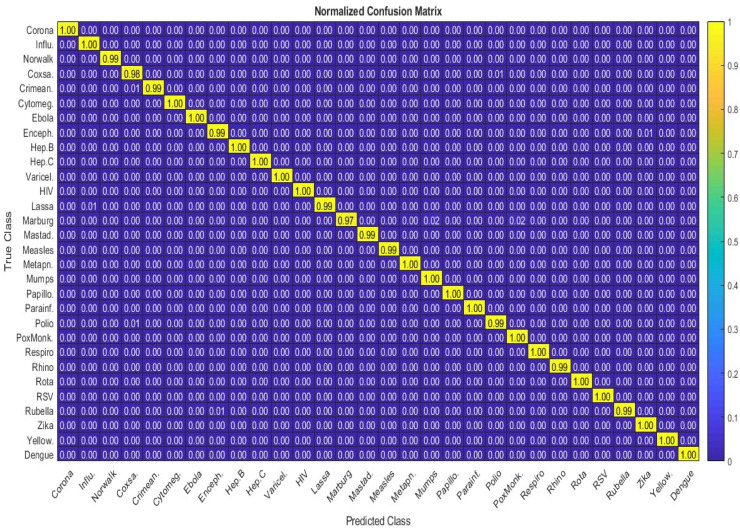
Obtained confusion matrix on 30-class problem.

The results shown in Fig. 18 indicates a very good evaluation of the proposed algorithm across all class types, with the lowest recorded F1 score being around 0.95, and the accuracy and AUC (area under curve of ROC) values for each class being above 0.97. However, it should not be forgotten that this dataset is highly imbalanced, which may pose challenges in training and tuning the parameters of the learning model for classes with very few samples. Additionally, in Fig. 19, the relevant confusion matrix is presented to show the percentage of correct predictions for each type of virus.

As shown in Fig. 19, the rows represent the actual class values, and the columns represent the predicted values. The values in the main diagonal of the matrix indicate the correctly predicted values for each class. The results for most classes are 100 % accurate, but there are some errors in the corresponding rows.

After designing the binary and multi-class models, additional tests were conducted on new data. One of these tests was an external evaluation of the trained model. New data related to the LP 8.1.1 coronavirus variant and the Nipah virus were downloaded from the NCBI website, consisting of 1278 and 51 samples, respectively. In the corresponding binary and three-class tests, they were successfully classified into the previously trained classes.

All of these experiments can also be adapted to binary classification tests, where a specific type of virus is distinguished from other viruses with very high accuracy. In this case, fewer features are needed to separate the types of viruses, and the influential centrality values for differentiating each virus from the others can be examined in greater detail. Additionally, the probabilities of input and output degrees for each type of node can also be analyzed separately. This feature extraction algorithm from genetic data not only provides very high accuracy for the classifier but is also related to the genetic nature of nucleotides and proteins.

The independence of the proposed approach's performance from the length or type of virus is one of its main advantages. In other words, for various reasons, samples of a specific virus may have nucleotide lengths that are shorter or longer. However, the proposed approach examines the complete structure of each genetic sample in textual form and converts it into a graph, thereby analyzing the chemical structure of each sample from a nucleotide perspective. Also, the rules extracted from each decision tree designed for each virus can provide meaningful results for genetics specialists or biologists on an individual basis.

The results indicated that certain specific features played a more significant role in distinguishing the output type. In other words, some triplets that were the focus of the research had a greater impact on the detection of COVID-19, with GTT being one of them. GTT (or GUU) triplet had previously been reported and discussed in [[48], [49], [50]], related to coronavirus codon usage bias concepts. According to the defined criteria in the graph domain, this suggests that these triplets can have a key role in influencing other triplets and the information dissemination process among different triplets.

Regarding the rules extracted from the second experiment, a number of new triplets demonstrated greater influence. Some of these, such as TGT, had been reported in [51]. However, others may have emerged in this experiment depending on the different viruses used during the training phase. This approach also led to the identification of several influential triplets in the multi-class experiment. Nevertheless, given the large number of classes, examining each type of virus in a binary classification framework may better reveal the distinguishing triplets.

In the second experiment, where the influenza virus was also included alongside coronaviruses, the role of certain codons in labeling influenza was more pronounced. For instance, the roles of the codons GGA and CTA were discussed in [52]. This indicates that the graph analysis of genetic samples may relate to the concepts of codon usage bias. However, some of the triplets presented in the literature on codon usage bias were not extensively discussed. This suggests that the relationship of certain nucleotide triplets may not only be global but can also influence the transmission or reception of information in specific cases. Additionally, depending on the samples in this study and other classes and viruses, some triplets may have become distinguishing features. Nevertheless, generally, each of the corresponding triplet values, depending on the type of virus and the binary or multi-class problem, requires further comparisons.

### 4.4 Comparison

This section is dedicated to comparing the proposed algorithm for feature extraction and classification of genetic data. Numerous studies have been conducted prior to this, each analyzing various aspects such as text format, signal processing, or using deep learning approaches with a black-box perspective. In the field of graph analysis, a series of investigations have also been carried out. All of this studies on genomic sequences classification scope are summarized in Table 5, which compare existing research. In this table, from left to right, the reference number, the number of research samples, the achieved accuracy (ACC), and the machine learning algorithm (ML) are listed. Each row contains the information for a specific article.

**Table 5:** Comparative analysis of genomic sequences classification approaches.

**#**	**Ref**	**Samples**	**Acc**	**ML**
1	[29]	66,153	93	CNN
2	[31]	40,353	97	BERT
3	[30]	191,456	96	CNN
4	[32]	347,162	98	MLP
5	[23]	100	99	XG Boost
6	[24]	1500,000	90	XG Boost
7	[39]	9100	94	Transformer
8	[33]	600,000	94	Graph Convolutional Network
9	[26]	82,802	98	CNN
10	*	173,228	99	DT

Table 5 compares the proposed approach with nine other articles in terms of machine learning methodology, sample size, and achieved accuracy. As indicated, some of these studies have conducted limited research on sample size. In others, the achieved accuracy is not very high, depending on the sample size or the classifier algorithm. Some machine learning algorithms, especially in deep learning scope, require more time and memory. The proposed approach also worked on the feature extraction phase and improving the feature space for the classifier, aiming to use a classifier with appropriate speed and time.

As indicated in Table 5, the proposed algorithm performed significantly better than other studies relative to the sample size and obtained accuracy of research. One of the advantages of using the proposed approach is the interpretability of the results, thanks to the use of a white-box decision tree model. However, it is important to note the lower time complexity of the decision tree compared to other algorithms. Additionally, it should be mentioned that the feature extraction algorithm of the proposed approach enabled high classification capabilities for samples of various types of viruses.

Furthermore, the large number of virus types included in this study indicates that the capabilities of this research are not limited to the coronavirus or any specific virus. In other words, this research demonstrated that the feature extraction approach used allows for the identification of characteristics based on the centrality of the feature vector, as well as the degree of input and output. For each type of virus, different nodes may be influential, but the accuracy of the resulting classification is very high.

### 4.5 Limitations and future works

The findings of the research can be further examined from two perspectives: artificial intelligence and genetics. From a computational standpoint, the proposed approach performed exceptionally well in the feature extraction phase and established a strong conceptual connection with influential nodes in the graph, as well as controlling the flow of incoming and outgoing information. In terms of graph centrality, each node has a specific influence, but understanding which connected nodes this influence pertains to, in relation to the rate of information flow, is also significant. The proposed approach took these factors into account and also included a series of genetic components that warrant further investigation. The question of whether one or more triplet nucleotides have a greater impact on the chemical structure of a specific type of virus can be thought-provoking. Given the concept of codons and the importance of triplet nucleotides, examining any specific virus through this lens could open up new perspectives in the fields of genetics and its chemical structure.

Given the interdisciplinary nature of the research topic, the challenges and limitations can be examined in two main categories. Regarding biological and genetic limitations, one can refer to the selected data and datasets. The data available on the NCBI website can be filtered based on various criteria, including cellular structure (type of DNA/RNA or type of realm). However, this also raises a series of challenging questions, such as the fact that the lengths of samples from different viruses are not uniform and come from various structural formats. Additionally, there is not an equal number of samples available for these types. In other words, for certain diseases and viruses, there are more samples in reputable datasets like NCBI for various reasons. This situation leads to an imbalance in the selected dataset despite the high diversity of viruses.

The proposed approach on genetic sequences can reveal a series of hidden features within the genetic structure of cells. These features as nucleotide triplets or codons or amino-acids, represented as nodes of graph, can hold significant meanings. Some of these concepts may not have been previously validated, and some may also be interrelated. This very issue raises questions within this field. However, there were also several challenges and issues that could be significant for future research in this area. The origin of these challenges may relate to the type of sampling and datasets used in this field, which could potentially be affected by laboratory errors or other factors impacting the genetic sequence data. Additionally, there are ambiguities or questions regarding genetic concepts that also influence the analysis of genetic data.

Other challenges related to computer science and machine learning scope. One of them is the issue of dataset imbalance. This can significantly affect the performance of certain machine learning models during the classification phase. It also impacts the validation phase and the selection of training and testing data. Nevertheless, this research aimed to employ appropriate validation approaches. In the classification phase, a decision tree model was used, which itself can present problems and challenges such as overfitting. Another problem is the feature extraction phase and the selection of influential features with the help of decision trees.

One of the challenges pertains to the features extracted from the decision tree. Given the dimensions of the graph and the number of nodes, a feature selection approach is employed to identify distinguishing features among different classes using the decision tree. However, the features comprise three graph metrics, which, if the goal is greater interpretability of the model, might yield better insights when used as three distinct features. In binary classification or cases with a low number of classes, this issue may not be as pronounced. However, in scenarios with a large number of classes and imbalanced mode, such as in this research 30-class problem test, this could be significant.

In this field, also future work can be classified into two sections: computational and biological aspects. In the biological domain, genetic data from other diseases or new variants of a virus can be tested. On the other hand, the interpretability of the results (in terms of codons or amino acids) for each virus can have connections with certain biological concepts. In the computational domain, new research can be conducted in various phases of the designed pattern recognition model.

In the feature extraction phase, various innovations can be utilized depending on the graphical perspective. If genetic data is viewed from a graph perspective, it is possible to extract other biological components that facilitate the differentiation or identification of specific types of viruses. Furthermore, the optimization of creating a hybrid feature from several graph metrics also raises questions. This topic could be explored further in the realm of graph metrics and optimization. Additionally, other interpretable approaches can be employed in the classifier phase. For future work, other rule-based or interpretable approaches could be tested in this phase to potentially yield meaningful results for the field of genetics. The use of modern deep learning techniques, such as language models, transformers, and graph neural networks, can also serve as a hybrid idea in this field.

## 5 Conclusion

Viral diseases such as influenza and coronaviruses have posed various challenges in different fields in recent years. Therefore, analyzing the internal structure of these viruses and others can play a significant role in addressing these challenges across various domains. This research conducted experiments on the genetic data of different viruses. Unlike most previous studies, it approached the analysis and classification of genetic data from a new perspective. The graph-based algorithms and artificial intelligence approaches used aimed to connect the research findings with genetic concepts within the cell.

In this research, the classification of genetic data and nucleotide sequences was examined from a different perspective. The use of graph algorithms enabled the extraction of new features with high interpretability from any type of virus. Modeling the intracellular genetic structure can be examined from two perspectives. From the biology and genetic perspective, there may be specific chemical properties between nucleotide triplets or nucleotides that warrant further investigation. On the other hand, using white-box machine learning approaches or other feature extraction methods that are rule-based may yield additional interpretable results.

In-degree, out-degree and eigen vector centrality features of a graph indicate meaningful concepts from the graph. This feature did not consider just the number of connections of a node, and the importance of connections are considered. These centrality measures could help prioritize nodes that are both central hubs of the network and connected to other major hubs, and potential key control points. The decision tree approach for classifying samples also effectively aligned with the research's goal of interpretability. The very high evaluation of the classification algorithm, with an accuracy exceeding 99 %, indicated this, and the rules derived from each experiment required interpretation. From a genetic standpoint, the utilized triplet structure of nucleotides necessitates further investigation into the extracted features.

Data availability

No datasets were generated and the datasets were obtained from the NCBI repository virus section (https://www.ncbi.nlm.nih.gov/labs/virus). The accession number of samples can be download from: https://github.com/aminkhodaei/DNA_sequence_database.

## CRediT authorship contribution statement

**Amin Khodaei:** Writing – original draft, Visualization, Validation, Resources, Project administration, Methodology, Data curation, Conceptualization. **Zahra Pourabbas:** Writing – original draft, Visualization, Methodology. **Fatemeh Hashem-zadehdizajyekan:** Writing – review & editing, Methodology, Conceptualization. **Erfan Esmaeili:** Validation, Data curation, Conceptualization.

## Declaration of competing interest

The authors declare that they have no known competing financial interests or personal relationships that could have appeared to influence the work reported in this paper.

## Data Availability

Data will be made available on request.
